# Retraction: Food insecurity and mental health of women during COVID-19: Evidence from a developing country

**DOI:** 10.1371/journal.pone.0349829

**Published:** 2026-05-21

**Authors:** The PLOS One Editors

After this article [1] was published, concerns were raised about the participant sampling method, the validity and reliability of the data analysis, the reproducibility of the results and the support for the conclusions. A detailed critique posted in [2] was used in *PLOS One*’s editorial assessment. PLOS followed up on the following specific concerns:

Specifically:

- In S1 Dataset, questions 4, 5, 7 and 8 in the Perceived Stress Scale (PSS) questionnaire are phrased in such a way that responses to these items would require reverse coding. The coding and analysis files underlying [1] are not available; however, the PSS mean reported in [1] is consistent with incorrect coding of these items (i.e., the reported results indicate that reverse coding was not applied where appropriate such that some answers indicating higher stress were coded as lower stress outcomes).
- There appear to be inconsistencies between the Food Insecurity Experience Scale (FIES) questionnaire results reported in Fig 1, S1 Dataset, and in the article text. The last author notified the journal that in the legend of Fig 1, Wave 1 and Wave 2 are erroneously switched; however, the *PLOS One* Editors consider that this does not fully resolve the inconsistences.
- The specific wording of the responses to the PSS questionnaire is reported differently in Table 3 and S1 Dataset.
- The last author confirmed that the sample of participants in [1] overlaps fully with the study population reported in a related study conducted by the same authors in [3,4] and partially with that in [5]. In response to this concern, the last author stated that this article [1] uses an observational baseline survey conducted before any intervention took place and participants were only re-used in a later study [3]. They acknowledged that participants in [5] were exposed to a public health awareness campaign during a time period that overlapped with conduct of the Wave 1 questionnaire in the *PLOS One* study [1].
- There appear to be some inconsistencies in the demographic data reported for those respondents with overlapping participant identity numbers across studies [1,3–5].
- There appear to be inconsistencies between the education and literacy variable within the participants recorded in [1], with 39 individuals recorded as both illiterate and having 8 or more years of education. The last author stated that such inconsistencies are not uncommon in large-scale phone surveys conducted under pandemic lockdown conditions. However, the *PLOS One* Editors consider that this does not fully resolve the inconsistences.

PLOS consulted a Statistical Advisor who agreed with the concerns raised, advising that the central conclusion relating to the association between food insecurity and stress is not adequately supported in light of these issues. They also noted that the high level of overlap between the participants in 3 separate published articles [1,3–5] raises concerns about the randomization process within the sampling methodology. They advised that without the underlying code used in the data analysis and further information on the sampling method, the research presented is not reproducible. The last author stated that the study does not involve randomized assignment or a randomly selected sample, but that the reference to randomization relates to a separate intervention context during the COVID-19 period and is orthogonal to the observational analysis presented in [1].

Further underlying data including the individual-level coding of the variables used in the analyses, code and analyses underlying Fig 1 and Tables 4 and 5 could not be obtained in editorial follow-up.

In light of the above unresolved concerns, which call into question the validity and reliability of the reported study design, results and conclusions, the *PLOS One* Editors retract this article.

TR and MDGH agreed with the retraction. AI did not agree with the retraction.
